# Evolution and stages of China’s economic inequality from 1978 to 2018

**DOI:** 10.1371/journal.pone.0288873

**Published:** 2023-07-20

**Authors:** Yuanzhi Guo

**Affiliations:** Institute of Geographic Sciences and Natural Resources Research, Key Laboratory of Regional Sustainable Development Modelling, Chinese Academy of Sciences, Beijing, China; Institute for Advanced Sustainability Studies, GERMANY

## Abstract

Regional economic inequality has long been a prominent problem in China’s national economic and social development. In this study, the centre of gravity (CG) model is employed to reveal the spatiotemporal patterns and stages of China’s economic inequality in 1978–2018, and the characteristics of different stages are explored. The results show that China’s economy achieved rapid growth at an average annual growth rate of 9.4% in 1978–2018. Due to the spatial heterogeneity of the location and the unbalanced development strategy, there was a significant economic gradient between eastern and western China. Spatially, there was a notable increase in the agglomeration of GDP and the added value of the three industries, and their CGs moved southwestward as a whole. According to the evolution of regional differentiation characteristics, combined with key historical events, China’s economic inequality from 1978 to 2018 could be divided into four stages, i.e., the stages of economic inequality led by institutional reform (1978–1991), market mechanisms (1992–2003), regional coordination (2004–2012), and socioeconomic transformation (2013–2018). The alternation of these four stages reflects how to scientifically deal with the relationship between efficiency and equity in economic development.

## 1 Introduction

Inequality is a common phenomenon in regional development. It not only pertains to the uniformity and nonuniformity of historical laws, but also features the spatial heterogeneity and diversity of historical trends [1, 2]. In economics, the spatial differences among locations cause certain economic activities to concentrate in specific areas with advantageous conditions for them, resulting in the spatial imbalance in economic performance, and the economic activities concentrated in a particular location will spill-over to other locations once they exceed the resource and environmental capacity of the original location [3, 4]. Therefore, economic inequality reflects the diversity and asymmetry of economic development and geography based on regional heterogeneity, and this imbalance has existed since the beginning of human society [1, 5]. In the sense that economic base determines the superstructure, widespread economic inequality has long been a focus of civil society and academic research, and reducing inequality within and between countries has become one of the key goals of global sustainable development.

Since the reform and opening-up in 1978, China has experienced rapid economic growth, but it also faces serious economic inequality and its associated challenges [6–9]. Nationally, this inequality mainly manifests itself as coastal-inland inequality [10, 11], east-west inequality [12–14], and urban-rural inequality [10, 15, 16], forming a gradient pattern of China’s economic development in different spatial dimensions. Due to the path dependence of economic development, these spatial patterns will be maintained in the long run [3, 17]. Additionally, there is also spatial heterogeneity within provinces, cities, and counties, such as the economic inequality between the Pearl River Delta (PRD) and the non-PRD regions of Guangdong [18, 19]. Temporally, the evolution of economic inequality describes the stages of China’s economic development [20, 21]. Empirical research shows that China was in the primary production stage in the 1980s, entered the early-mid stage of industrialization in the first decade of the 21st century [22], and entered the post-industrialization stage at the end of the second decade of the 21st century [23].

In terms of the mechanisms behind economic inequality, there is a broad consensus across disciplines that "locational autocracy" is the main cause [4]. The spatial heterogeneity in physical geography, unbalanced development strategies and the resulting inequalities in production, distribution and consumption are the main influencing factors of regional economic inequality in China [3, 4, 24, 25]. The heterogeneity between urban and rural areas is the basis of urban-rural economic inequality [26], and the solidified urban-rural dual structure based on the household registration (*hukou*) system is the root cause of China’s widening urban-rural gap [15, 27, 28]. These factors have shaped decisions related to regional economic development, but this has changed considerably with technological advances. In addition to traditional factors such as population and land, globalization [29–31], high-speed railway [32, 33], internet technologies [34] and other emerging factors are continuing to reshape China’s regional economic patterns. Under the influence of economic inequality, spatial imbalances in the allocation of resources such as infrastructure and public services, as well as development achievements such as social welfare and personal income, are increasingly evident in different regions [35, 36], which further leads to increasingly uneven flows of population, capital and other factors [37–39]. If economic inequality is allowed to develop arbitrarily, it is not conducive to the improvement of resource allocation efficiency and sustainable socio-economic development [25, 40, 41]. To reverse this situation, the Chinese government has implemented a series of strategies to accelerate the development of central and western China, mainly including the Western Development, the Revitalization of Northeast China, and the Rise of Central China. Guided by these national major strategies, local governments have also issued a series of policies and measures to promote coordinated development within the region [42, 43].

Economic inequality in China is an important research object of economics, management, and other disciplines, which focus on exploring the causes and mechanisms behind it, analyse its negative impact on socio-economic development, and propose countermeasures. Although studies on the stages of economic inequality in China are relatively scarce, there are more studies focusing on the stages of economic growth in China [22, 44–46]. Drawing on the classical theories of economics such as Chenery’s economic development theory and Rostow’s stage theory of economic development, many scholars have extensively discussed the criteria for classifying China’s economic development stages, forming structuralist, aggregationist and synthesisist views [47]. In general, most of these studies are based on empirical judgments and lack the support of quantitative models, which compromises the scientific nature of the research results [44, 48].

Currently, China’s economy has entered a new normal stage, with more emphasis on quality rather than quantity of economic development. Against this background, the Chinese government has proposed to implement the regional coordinated development (RCD) strategy and accelerate the construction of a new development pattern. Scientific understanding of the stage of economic development and its characteristics is an important basis for a country to formulate economic policies [46]. Therefore, a systematic analysis of the evolution and stages of China’s economic inequality is significance for the formulation of RCD policies. Using a dataset of China’s economic development in 1978–2018, this study quantitatively analyses the spatial and temporal patterns of China’s economic inequality, divides the stages of China’s economic inequality according to the evolution of economic inequality and key historical events, and discusses its causes and characteristics at different stages. The findings will provide scientific support for promoting RCD and validating the existing studies on the division of China’s economic development stages.

## 2 Materials and methods

### 2.1 Materials

Economic inequality is a broad concept that refers to inequality in the distribution of income and other economic factors, such as wealth, employment, or human capital [49]. In this study, GDP, a measure of economic performance, is employed to investigate China’s economic inequality. To comprehensively understand the evolution of China’s economic inequality from 1978 to 2018, this study makes full use of national and provincial GDP data. Specifically, data on GDP and the added-value of primary, secondary and tertiary industries at the national and provincial levels are collected from the China Statistical Yearbook 2019 (http://www.stats.gov.cn/sj/ndsj/2019/indexeh.htm) and the provincial statistical yearbooks of previous years, respectively. Other economic data used in the subsequent analysis is also drawn from these statistical yearbooks. Demographic data come from the China Population Statistics Yearbook (https://data.cnki.net/yearBook/single?id=N2022040097). Data on administrative divisions come from the Standard Map Service Platform (http://bzdt.ch.mnr.gov.cn/). In line with the research design, Hong Kong, Macao, and Taiwan are excluded from the analysis. Therefore, a total of 31 provincial units are obtained to explore the spatial inequality and stages of China’s economic growth.

Furthermore, the Chinese mainland is divided into four regions based on social and economic differences, i.e., eastern China, central China, western China, and north-eastern China (http://www.stats.gov.cn/ztjc/zthd/sjtjr/dejtjkfr/tjkp/201106/t20110613_71947.htm). Specifically, eastern China includes Beijing (BJ), Tianjin (TJ), Hebei (HeB), Shandong (SD), Jiangsu (JS), Shanghai (SH), Zhejiang (ZJ), Fujian (FJ), Guangdong (GD) and Hainan (HaN); central China includes Shanxi (SX), Henan (HeN), Anhui (AH), Hubei (HuB), Hunan (HuN) and Jiangxi (JX); western China includes Guangxi (GX), Guizhou (GZ), Chongqing (CQ), Sichuan (SC), Yunnan (YN), Tibet (XZ), Shaanxi (ShX), Gansu (GS), Qinghai (QH), Ningxia (NX), Xinjiang (XJ) and Inner Mongolia (NMG); and northeastern China includes Heilongjiang (HLJ), Jilin (JL) and Liaoning (LN).

### 2.2 Methods

Due to the zonality of natural and human elements, the spatial distribution of human activities on the earth’s surface is characterized by a clear regional differentiation. The centre of gravity (CG) refers to the point in an object where the weight is evenly dispersed and the object is balanced in all directions. In addition to the shape, the position of the CG is determined by the mass distribution of the object. This feature determines that CG can not only reveal the overall inequality of the research object, but also describe the regional differentiation characteristics of the research object [50, 51]. To examine the regional divergence of China’s economic growth from 1978 to 2018, this study calculates the economic CG, including the CG of GDP and the CG of the added-value of primary industry, secondary industry, and tertiary industry. In general, the CG is given as follows [50]:

$${\bar{X}}_{a}=\sum_{i=1}^{n}w_{i}x_{i}/\sum_{i=1}^{n}w_{i};\;{\bar{Y}}_{a}=\sum_{i=1}^{n}w_{i}y_{i}/\sum_{i=1}^{n}w_{i}$$ (1)

where (${\bar{X}}_{a},\;{\bar{Y}}_{a}$) is the coordinate of the weighted average centre of each spatial unit in year *a*, (*x*_*i*_, *y*_*i*_) represents the geographic coordinate of spatial unit *i*, *n* refers to the number of spatial units, and *w*_*i*_ refers to the weight of spatial unit *i*. Supposing that *P*_*k*_ (*x*_*k*_, *y*_*k*_) and *P*_*k+m*_ (*x*_*k+m*_, *y*_*k+m*_) are the coordinates of economic CG in years *k* and *k+m*, respectively, the distance between the two centres can then be calculated as follows:

$$D_{km}=C\times \sqrt{{\left(x_{k+m}-x_{k}\right)}^{2}+{\left(y_{k+m}-y_{k}\right)}^{2}}$$ (2)

where *D*_*km*_ denotes the distance; *C* is a coefficient that refers to the constant for converting the unit of geographical coordinates to horizontal distance, with a value of 111.1 km.

## 3 Results

### 3.1 China’s economic growth from 1978 to 2018

**Fig 1:**
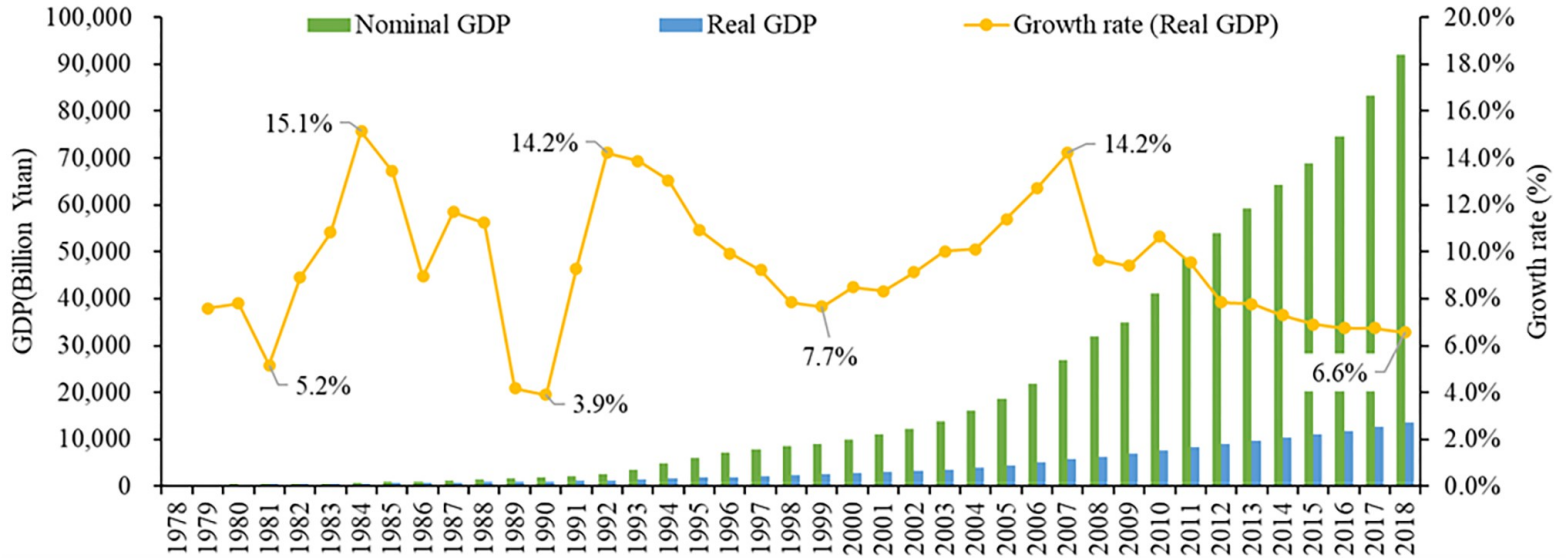
Evolution of GDP in China from 1978 to 2018. Note: Real GDP is calculated at the constant price of 1978.

Driven by institutional reforms and policy innovations, China has continued to improve its market system, optimize resource allocation and promote economic growth. In 1978, when the reform and opening-up policy was released, China was one of the poorest countries in the world, with a GDP of 367.9 billion yuan and a per capita GDP of 385 yuan. In 2018, China’s GDP increased to 91.9 trillion-yuan, accounting for approximately 16% of world GDP, and the per capita GDP increased to 64,644 yuan, equivalent to $9,780. If calculated at the constant price of 1978, China’s GDP in 2018 was 13.5 trillion yuan, with an average annual growth rate of 9.4% in 1978–2018 (Fig 1). During this process, the share of the added-value of the primary sector in GDP decreased from 27.7% to 7.0%, while that of tertiary sector increased from 24.6% to 53.3%. The proportion of the added-value of the secondary industry remained relatively stable over time and has gradually declined in recent years, from 47.7% in 1978 to 46.5% in 2011 and then to 39.0% in 2018. These changes indicate that the tertiary sector has replaced the secondary sector as the main force driving China’s economic growth. Meanwhile, China has basically completed industrialization and is gradually moving toward the post-industrialization stage.

**Fig 2:**
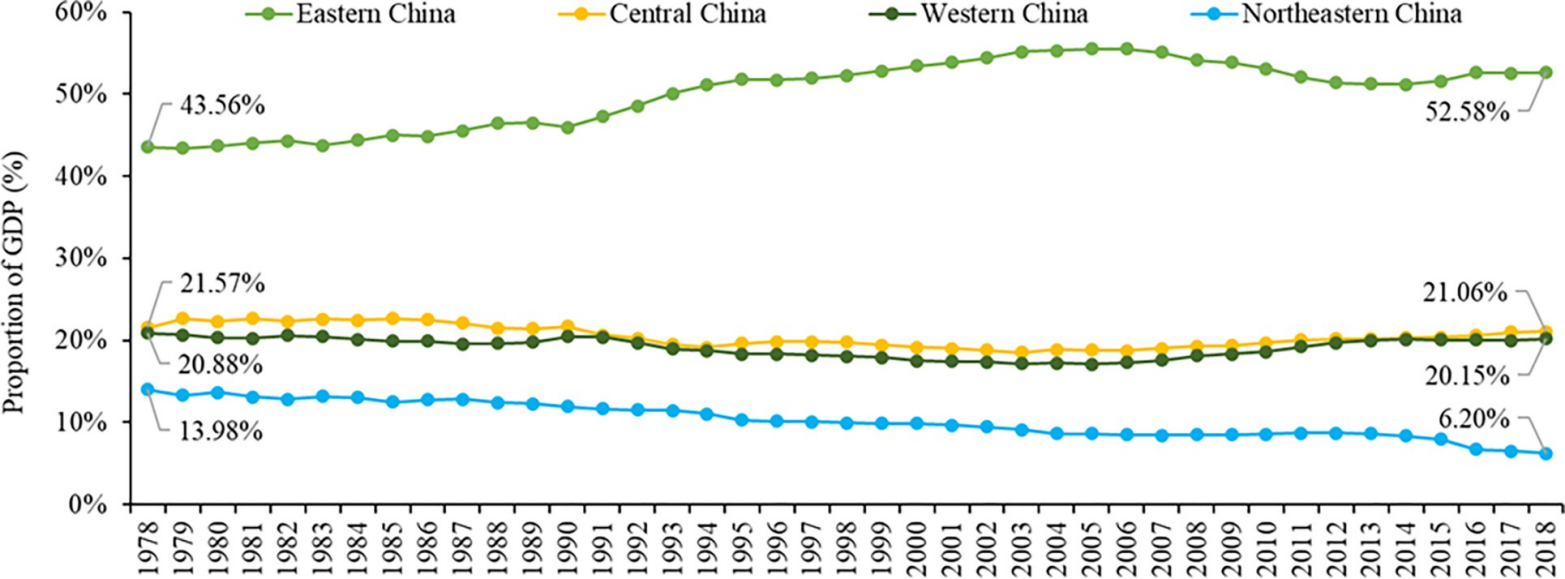
The proportion of GDP in China’s four regions from 1978 to 2018.

In terms of the four regions, the eastern region’s share in the national GDP increased rapidly, from 43.6% in 1978 to 52.6% in 2018, with the highest value of 55.5% in 2006. In north-eastern China, this share fell sharply from 14.0% to 6.2%. In central and western China, its share remained stable, declining only from 21.6% and 20.9% to 21.1% and 20.1%, respectively (Fig 2). The eastern region generated nearly half of the country’s GDP with less than 10% of the land, while the western region generated about 20% of the country’s GDP with over 70% of the land. Furthermore, an analysis of the industrial structure of the four regions showed a rapid decline in the added-value of the primary sector as a share of GDP, from 23.3%, 39.2%, 36.9% and 20.0% to 4.6%, 8.4%, 11.0% and 10.9% in eastern, central, western and north-eastern China, respectively. The share of the added-value of secondary sector remained relatively stable in the central and western regions, with a value of about 42%, but dropped sharply in the eastern and north-eastern regions, from 56.8% and 64.3% to 40.8% and 36.1%, respectively. The share of the added-value in the tertiary sector increased substantially from 19.8%, 18.4%, 19.8% and 15.7% to 54.6%, 47.6%, 48.5% and 53.0%, respectively. Therefore, during the period of 1978–2018, the dominant industries in the four regions all changed from secondary industry to tertiary industry, especially in eastern and north-eastern China.

### 3.2 Evolution of China’s economic inequality from 1978 to 2018

**Fig 4:**
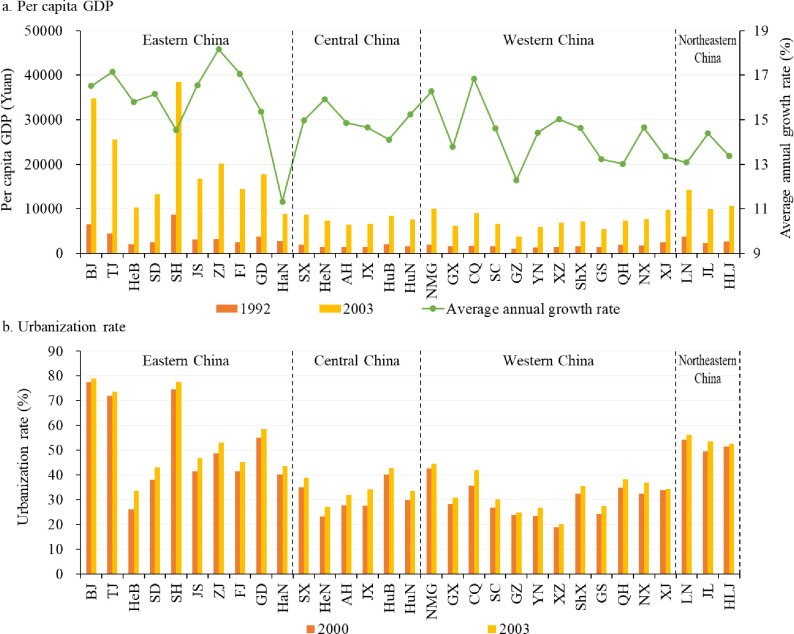
The development of provincial per capita GDP (a) and urbanization rate (b) from 1992 to 2003. Note: Due to the availability of data, the years selected for the urbanization rate for resident population are 2000 and 2003.

The movement of CG can reflect the changes in the spatial pattern of geographical objects/phenomena [50]. Therefore, the CG of provincial GDP and added-value of the three industries from 1978 to 2018 were calculated, and spatial visualization was employed to reveal the evolution of the spatial pattern of China’s economic inequality (Fig 4). Most CGs were concentrated in the eastern and southern part of Henan Province, and only a small portion distributed in southwestern Shandong Province and north-western Anhui Province. The starting-ending point analysis shows that the CG of GDP in 1978 was in Zhecheng County of Henan Province and moved to Zhengyang County of Henan Province in 2018, and the CG of the added-value of primary, secondary and tertiary sectors moved from Biyang County of Henan Province, Shanxian County of Shandong Province and Huaiyang County of Henan Province to Tanghe County, Luoshan County, and Xixian County of Henan Province, respectively (Table 1). In additional, Eq (2) was employed to measure the moving distance of economic CG. The results showed that the distance between the CG of GDP in 1978 and 2018 was 188.7 km, and the distance between the CG of the added-value of primary, secondary and tertiary industries in 1978 and 2018 was 76.6 km, 329.8 km and 126.5 km, respectively.

**Table 1:** The location of China’s economic CG in starting and ending years and its moving distance.

	GDP	Added-value of primary industry	Added-value of secondary industry	Added-value of tertiary industry
**1978**	Zhecheng County (115.12E, 33.99N)	Biyang County (113.57E, 32.80N)	Shanxian County (116.12E, 34.79N)	Huaiyang County (114.78E, 33.64N)
**2018**	Zhengyang County (114.62E, 32.58N)	Tanghe County (112.74E, 32.72N)	Luoshan County (114.46E, 32.20N)	Xixian County (114.75E, 32.52N)
**Distance**	188.7 km	76.6 km	329.8 km	126.5 km

**Fig 3:**
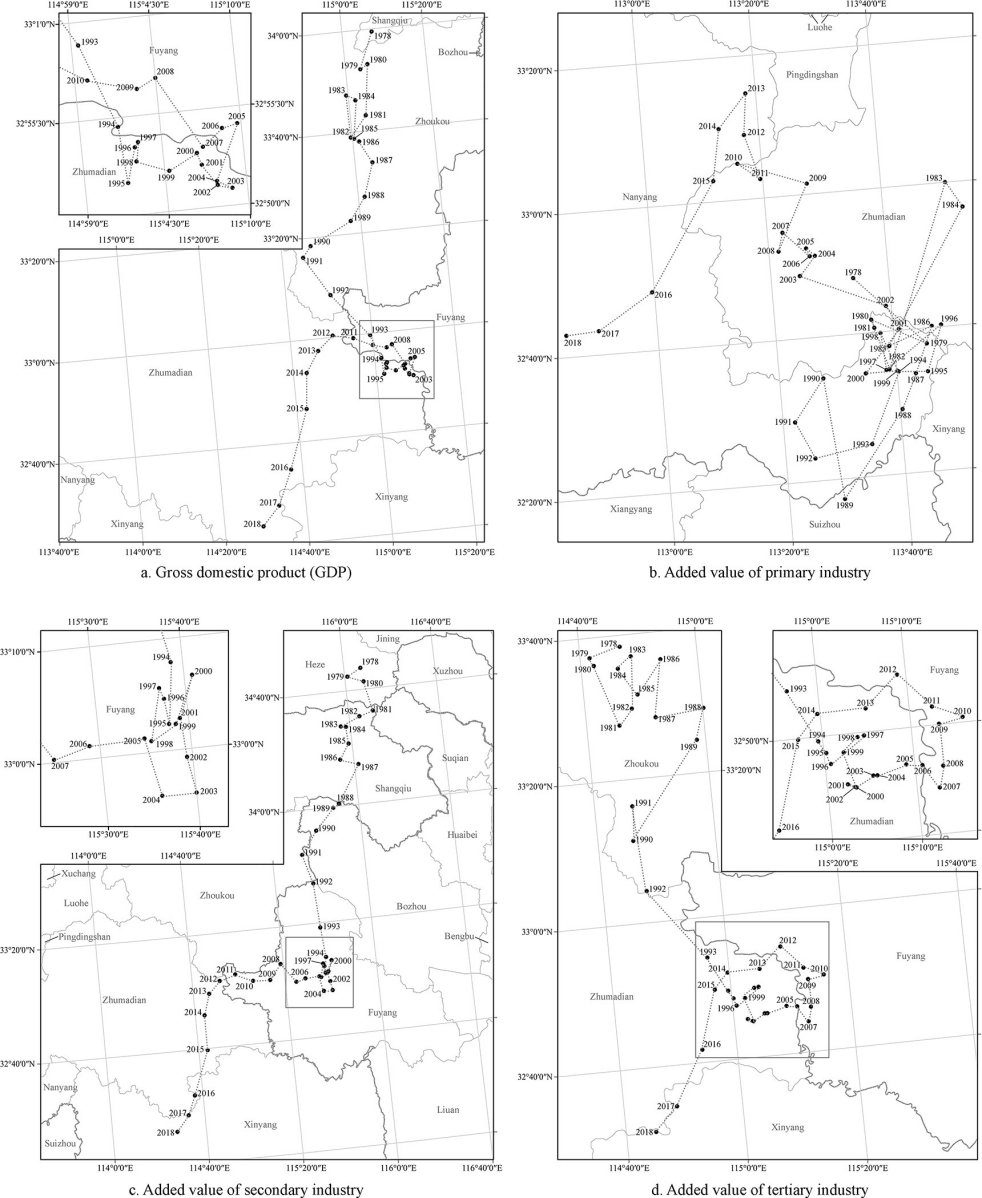
Spatial movement of the economic CG in China from 1978 to 2018. (The source of map: http://bzdt.ch.mnr.gov.cn/browse.html?picId=%224o28b0625501ad13015501ad2bfc0265%22).

The analysis of the moving direction of economic CG showed that the CG of GDP and the added-value of the three industries generally moved in a southwestern direction from 1978 to 2018, but there were obvious differences in the specific moving direction (Fig 3). The CG of GDP moved to the southwest in 1978–1991, to the southeast in 1992–2003, and to the northwest and southwest in 2004–2012 and 2013–2018, respectively. In terms of the CG of the added-value of the three industries, the moving direction of the CG of the added-value of primary industry had no obvious regularity until 2001, showing a characteristic of moving to the northwest in 2002–2013 and moving to the southwest in 2014–2018. The moving direction of the CG of the added-value of secondary industry was similar to that of GDP. Specifically, the CG of the added-value of secondary industry shifted to the southwest in 1978–1991, to the southeast in 1992–2003, and to the northwest and southwest in 2004–2011 and 2012–2018, respectively. The CG of the added-value of tertiary industry showed a southward movement in 1978–1992, a southeastward movement in 1993–2002, then a northward movement after an eastward movement in 2003–2007, and a southwestward movement in 2013–2018.

### 3.3 Stages of China’s economic inequality from 1978 to 2018

Economic growth is the result of a combination of natural and human factors in a region. Due to the relative stability of natural factors and the drastic changes in human factors, economic inequality across regions arises from policy and institutional orientations at different times [52]. Based on the evolution of the economic CG and combined with key historical events, this study divides China’s economic inequality in 1978–2018 into four stages and names them as the stage of economic inequality led by institutional reform (1978–1991), the stage of economic inequality led by market mechanism (1992–2003), the stage of economic inequality led by regional coordination (2004–2012), and the stage of economic inequality led by socioeconomic transformation (2013–2018).

#### 3.3.1 Stage of economic inequality led by institutional reform (1978–1991)

In 1978, the Third Plenary Session of the Eleventh Central Committee of the Communist Party of China (CPC) initiated the policy of reform and opening-up, aiming to improve the top-level design through measures such as system reform and policy innovation. These reforms adjusted and optimized production relations, broke the shackles of the previous system that limited economic growth, as well as liberated and developed the productive force. The reform efforts began in rural areas, where the household responsibility system (HRS) played a significant role in motivating farmers to produce and promoting agricultural development [53]. However, due to poor foundation, unstable policy expectations, and agriculture’s own weakness, the development of primary industry fluctuated in various regions, which was directly reflected in the irregular movement in the CG of the added-value of primary industry. The opening-up policy based on comparative advantages concentrated limited resources in specific regions to form economic growth poles and drive the development of the whole country through its spill-over effect. China formed a gradient opening-up pattern of special economic zones, coastal open cities, and coastal economic open zones in the early 1990s. In this process, labour-intensive and export-oriented processing industries developed rapidly in south-eastern areas, particularly in the Pearl River Delta, where the local governments took full location advantages of its proximity to Hong Kong and Macao to develop the “three-plus-one” trading mix. As a result, economic growth in Guangdong was considerably faster than that in other provinces (Table 2), and the CG of the added-value of secondary industry continued to move in a southwestern direction. The agglomeration of factors such as population and industry also promoted the development of the service industry in the frontier areas of reform and opening-up. However, the development of the tertiary industry showed obvious volatility due to the conflict and friction between the planned economy and market economy. Therefore, the CG of the added-value of tertiary industry moved southward in fluctuation. Due to the dominant position of secondary industry in the national economic development, the CG of GDP during this period moved towards the southwest.

**Table 2:** Statistics of the average annual growth rate of GDP in different provinces and regions.

Province	1978–1991				1992–2003				2004–2012				2013–2018			
	Primary industry	Secondary industry	Tertiary industry	GDP	Primary industry	Secondary industry	Tertiary industry	GDP	Primary industry	Secondary industry	Tertiary industry	GDP	Primary industry	Secondary industry	Tertiary industry	GDP
**Eastern China**	**6.06%**	**10.74%**	**13.31%**	**10.20%**	**5.01%**	**14.81%**	**12.99%**	**12.82%**	**4.19%**	**12.80%**	**12.22%**	**12.01%**	**3.05%**	**6.34%**	**9.07%**	**7.48%**
**Beijing**	6.86%	7.73%	12.82%	9.19%	1.48%	10.16%	13.24%	11.38%	1.26%	9.20%	11.06%	10.60%	-5.62%	5.07%	7.44%	6.88%
**Tianjin**	7.38%	6.95%	9.15%	7.58%	5.79%	13.01%	12.83%	12.47%	3.20%	17.70%	13.55%	15.87%	2.25%	5.84%	8.36%	7.12%
**Hebei**	4.98%	8.65%	12.76%	8.67%	6.17%	13.97%	11.94%	11.94%	4.38%	12.97%	12.02%	11.59%	3.34%	4.38%	10.38%	6.66%
**Shanghai**	2.03%	6.76%	10.30%	7.42%	2.87%	12.20%	13.65%	12.31%	-1.73%	9.06%	11.16%	10.14%	-5.69%	2.82%	9.04%	6.86%
**Jiangsu**	4.63%	12.07%	14.66%	10.82%	4.52%	14.83%	13.52%	12.97%	4.12%	13.59%	13.36%	12.84%	1.83%	7.08%	9.12%	7.78%
**Zhejiang**	4.32%	15.94%	13.52%	12.18%	4.49%	16.39%	13.14%	13.72%	2.76%	10.91%	12.64%	11.14%	2.04%	6.30%	9.31%	7.62%
**Fujian**	6.86%	13.68%	14.46%	11.71%	6.28%	17.08%	12.09%	13.12%	4.00%	15.77%	11.94%	13.05%	3.78%	8.26%	10.15%	8.74%
**Shandong**	7.32%	11.37%	12.87%	10.32%	5.06%	14.92%	13.49%	12.69%	4.44%	13.90%	12.91%	12.69%	3.62%	6.91%	9.02%	7.62%
**Guangdong**	7.64%	15.22%	16.21%	13.12%	3.57%	16.67%	12.93%	13.65%	4.17%	12.70%	11.30%	11.66%	3.48%	6.68%	8.64%	7.52%
**Hainan**	8.51%	10.91%	13.28%	10.42%	9.53%	11.55%	7.92%	9.24%	7.10%	15.82%	13.02%	11.99%	4.34%	5.90%	9.13%	7.32%
**Northeastern China**	**4.92%**	**6.53%**	**13.55%**	**7.69%**	**5.57%**	**10.26%**	**10.29%**	**9.54%**	**5.90%**	**15.16%**	**11.76%**	**12.77%**	**3.48%**	**2.48%**	**6.81%**	**4.50%**
**Liaoning**	5.05%	6.36%	14.70%	7.97%	6.31%	10.16%	10.14%	9.69%	5.69%	15.86%	10.67%	12.83%	1.57%	1.42%	5.33%	3.19%
**Jilin**	6.27%	7.90%	13.18%	8.69%	5.98%	11.23%	11.15%	10.00%	5.17%	16.79%	13.73%	14.04%	3.72%	5.15%	7.43%	5.90%
**Heilongjiang**	3.90%	6.17%	12.13%	6.78%	4.53%	9.86%	9.94%	8.99%	6.69%	12.58%	12.10%	11.73%	5.04%	2.34%	8.59%	5.70%
**Central China**	**4.69%**	**10.11%**	**13.28%**	**8.83%**	**4.86%**	**13.03%**	**11.89%**	**10.74%**	**4.76%**	**15.83%**	**11.65%**	**12.71%**	**3.88%**	**7.70%**	**9.88%**	**8.15%**
**Shanxi**	2.97%	7.73%	11.71%	7.91%	3.75%	12.01%	11.87%	11.16%	3.01%	12.82%	9.94%	11.12%	2.73%	2.98%	8.11%	5.25%
**Anhui**	2.91%	12.28%	13.73%	8.47%	5.15%	12.73%	14.13%	11.36%	4.62%	17.51%	10.95%	13.01%	3.76%	8.60%	9.98%	8.62%
**Jiangxi**	6.22%	10.10%	11.86%	8.94%	4.30%	13.18%	11.21%	9.84%	4.90%	16.35%	10.55%	12.66%	4.12%	9.06%	10.22%	9.06%
**Henan**	5.59%	10.96%	16.00%	9.98%	6.16%	14.00%	10.92%	11.27%	5.12%	14.99%	12.00%	12.58%	4.04%	7.76%	9.88%	8.14%
**Hubei**	4.70%	10.82%	13.41%	9.21%	4.26%	12.60%	12.01%	10.54%	4.84%	16.35%	12.73%	13.32%	3.96%	8.01%	10.02%	8.46%
**Hunan**	4.55%	8.25%	11.10%	7.74%	4.24%	12.86%	11.51%	9.95%	4.53%	16.29%	12.44%	12.93%	3.70%	7.40%	10.46%	8.36%
**Western China**	**6.32%**	**8.70%**	**12.76%**	**8.88%**	**4.77%**	**12.11%**	**12.23%**	**10.22%**	**5.17%**	**17.21%**	**12.22%**	**13.46%**	**4.56%**	**7.97%**	**8.99%**	**8.10%**
**Inner Mongolia**	7.78%	7.75%	14.62%	9.60%	5.11%	13.45%	14.51%	11.82%	5.43%	22.09%	14.34%	17.05%	3.12%	6.07%	7.08%	6.39%
**Guangxi**	5.66%	9.34%	8.49%	7.54%	6.17%	13.88%	10.82%	10.52%	5.55%	18.06%	11.88%	13.29%	4.20%	7.08%	9.06%	7.56%
**Chongqing**	6.06%	8.70%	11.91%	8.47%	3.18%	13.07%	12.52%	10.72%	4.58%	18.26%	12.47%	14.44%	4.42%	9.46%	10.30%	9.56%
**Sichuan**	5.66%	9.52%	12.21%	8.64%	5.15%	11.93%	12.12%	10.13%	3.98%	18.40%	11.19%	13.34%	3.74%	7.80%	9.46%	8.06%
**Guizhou**	6.45%	9.95%	12.21%	9.22%	3.12%	11.73%	10.61%	8.90%	4.78%	14.12%	13.84%	12.48%	6.46%	10.92%	10.78%	10.26%
**Yunnan**	5.66%	9.75%	13.92%	9.50%	4.23%	10.11%	12.38%	9.38%	6.01%	14.42%	11.59%	11.93%	6.02%	9.71%	8.72%	8.78%
**Tibet**	5.68%	1.81%	13.29%	7.15%	3.88%	19.80%	18.37%	13.30%	3.97%	17.27%	12.26%	12.33%	3.98%	14.32%	8.38%	10.20%
**Shaanxi**	6.23%	8.69%	15.03%	9.38%	3.28%	13.60%	10.76%	10.70%	6.28%	16.13%	12.59%	13.82%	4.40%	8.25%	9.18%	8.30%
**Gansu**	6.29%	6.12%	13.44%	8.09%	4.60%	10.34%	13.15%	10.24%	5.68%	13.52%	11.59%	11.61%	5.36%	5.18%	8.59%	6.88%
**Qinghai**	4.50%	5.81%	8.25%	6.34%	2.18%	12.04%	10.45%	9.55%	4.72%	15.62%	10.63%	12.59%	5.02%	8.38%	8.04%	7.98%
**Ningxia**	6.91%	7.78%	12.12%	8.88%	5.17%	11.62%	10.51%	9.76%	6.07%	15.64%	9.73%	12.19%	4.60%	7.88%	8.14%	7.78%
**Xinjiang**	9.76%	9.41%	15.74%	10.99%	6.07%	8.52%	11.75%	8.87%	6.31%	12.59%	11.76%	11.02%	5.42%	7.04%	9.89%	8.01%

Note: Data in this table are calculated at the constant price of 1978.

#### 3.3.2 Stage of economic inequality led by market mechanisms (1992–2003)

During Deng’s southern tour inspection in 1992, he emphasized the need to deepen reforms and accelerate economic development, marking a new stage for China’s reform and opening-up. The 14th National Congress of the CPC held in the same year determined that the goal of domestic reform was to establish a socialist market economy. Therefore, the Chinese government actively promoted the reform of the financial system, fiscal and taxation system, investment and financing mechanisms and other institutions, giving full play to the fundamental role of market mechanism in resource allocation. Meanwhile, the opening-up policy continued to expand to the cities along rivers, inland areas and border areas, basically forming a comprehensive, broad and multi-level opening-up pattern. The government also continued to concentrate its limited resources in advantageous areas to further develop economy. Against this background, production factors such as talents, capital, science and technology continued to gather in the eastern coastal areas with superior locations such as good economic foundation and relatively sound market mechanisms. The Pearl River Delta and Yangtze River Delta were at the forefront of industrialization and urbanization (Fig 4), resulting in the provinces in these regions developing their primary and secondary industries much faster than those in other regions. As a result, the CG of the added-value of primary and secondary industries moved towards the southeast in this stage. However, rapid industrialization and urbanization occupied a significant amount of cultivated land and agricultural spaces, especially in the south where per capita cultivated land was insufficient [54–56].

Accordingly, the CG of the added-value of primary industry moved northward as a whole. Nevertheless, due to the dominant position of the non-agricultural industry in economic development, the CG of GDP showed a trend of moving towards the southeast from 1992 to 2003.

#### 3.3.3 Stage of economic inequality led by regional coordination (2004–2012)

The unbalanced development strategy widened the economic gap between eastern and central-western China. In response, the Chinese government launched the western development strategy in 2000. However, due to factors such as poor industrial foundations and low environmental carrying capacity, the economic development of the vast western region was slow in the early stage of the strategy [57]. In 2004, the proposal of the revitalization of old industrial bases in north-eastern China and the rise of central China built up a complete RCD strategic system in China. Due to the rapid industrialization and urbanization, the eastern region experienced rising costs of land, energy, labour, and environmental protection [58], which greatly compressed the development space of traditional industries and led to the need for industrial transformation and upgrading. Traditional industries such as labour-intensive and resource-intensive industries in the Yangtze River Delta and Pearl River Delta moved to the central and western regions on a large scale. However, the cultivation of new industries took time. Meanwhile, infrastructure construction promoted by the RCD strategy required a large number of industrial products, leading to the rapid growth of heavy and chemical industries in the northern economy. As a result, the CG of the added-value of secondary industry generally moved to the northwest. The continuous adjustment of the industrial structure in the eastern region promoted the development of the service industry, which gradually replaced the secondary industry as the new driver of regional economic growth [59]. The CG of the added-value of tertiary industry in China moved eastward and northward. In terms of the primary industry, preferential policies such as the abolition of agricultural taxes and fees, agricultural subsidies and new countryside construction promoted agricultural development, and the government provided strong supports for major grain-producing areas to address food security concerns. As China’s major grain-producing areas were primarily in the north, the CG of the added-value of primary industry moved to the northwest. Thus, the RCD, which mainly focused on industrial transfer and preferential policies, shifted the CG of GDP to move to the northwest.

#### 3.3.4 Stage of economic inequality led by socioeconomic transformation (2013–2018)

**Fig 5:**
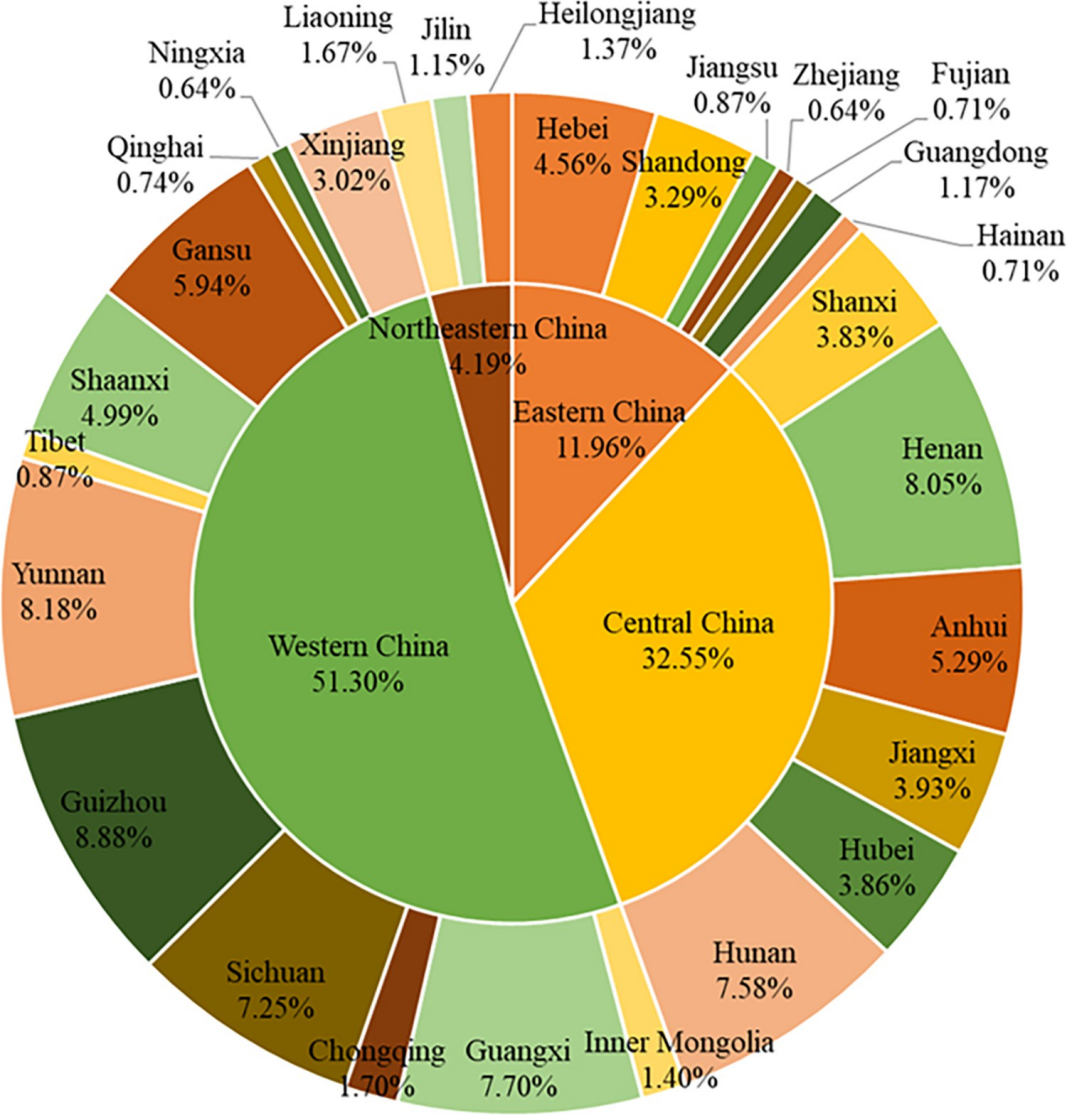
Spatial distribution of China’s rural poverty-stricken population in 2014.

The report of the 18th National Congress of the CPC declared that China had entered a critical phase of building a moderately prosperous society in all respects. To achieve this goal, China needed to address its shortcomings and accelerate the development of its underdeveloped central and western regions. Particularly, there was an urgent to ensure that the impoverished population in these areas did not lack food and clothing, and that their needs for compulsory education, basic healthcare and safe housing were guaranteed [60]. In response, the Chinese government introduced the strategy of targeted poverty alleviation to transfer talent, capital, technology and other resources to 22 provinces in central and western China, and to accelerate infrastructure development such as transportation and water conservancies. As a result, poor areas saw significant improvements in their endogenous growth dynamics, contributing to production development, ecological protection and living improvement [61]. Spatially, the poverty-stricken population in rural China was concentrated in western and central China (Fig 5). The increasing resource input had a greater impact on economic development in these regions than in other regions. Additionally, the deepening of China’s reform and opening-up pushed the country’s economic development to a new normal stage. Driven by various pressures such as the financial crisis and rising costs, the industrial transformation and upgrading in eastern China progressed steadily; the economic transformation in north-eastern China was hindered by rigid institution, macroeconomic downturn and irrational industrial structure; the central and western regions realized the optimization of industrial structure and rapid economic development through the mutual promotion of poverty alleviation and socioeconomic development. Table 2 shows that economic growth in central and western China was significantly faster than that in eastern China, while the development in north-eastern China was slow. Accordingly, the CG of GDP and the added-value of the three industries moved toward southwest in 2013–2018.

## 4 Discussion

Economic inequality is essentially based on uneven regional economic development. Therefore, the key to exploring the evolution and stages of economic inequality lies in analysing the changing trajectory of economic development and revealing the external manifestations of substantial transitions in the content of economic development [46]. Clark [62] once pointed out that economic development usually involves eight aspects: production expansion, technological progress, industrial structure transformation, capital accumulation, international economic relations adjustment, demand structure upgrading, institutional improvement and value change. The diversity of economic development content determines the criteria for measuring economic development are multidimensional. Correspondingly, the criteria and specific contents of the classification of China’s economic development stages are diversified [22]. However, whichever criterion is chosen, a common principle must be followed is the selection of events that have a decisive impact on economic development as milestones [63]. From this point of view, the time points selected in this study when exploring the evolution and stages of China’s economic inequality from 1978 to 2018 are consistent with most existing research on economic development stages [46, 63–65].

Due to the spatial heterogeneity of location, the opportunities and conditions for economic development are unequal across regions [3, 4]. Regions with superior locations tend to achieve economic growth more easily because they have better access to resources and market opportunities, while regions lacking these conditions may face economic stagnation and backwardness. Therefore, economic inequality is an inevitable phenomenon that exists not only in underdeveloped countries but is also widespread in developed countries [66–68]. Meanwhile, the costs of economic inequality are large and diverse [69], and this has become an important constraint to regional sustainable development. When economic inequality increases, the duration of economic growth tends to be shorter, which in turn affects economic growth in the medium to long term [70]. It is estimated that in OECD countries such as the United States and the United Kingdom, economic growth would have been 6 to 9 percentage points higher over the past 20 years without an increase in economic inequality [71].

In the early stage of reform and opening-up, China implemented a differentiated, focused, and selective unbalanced development strategy to develop its economy with limited resources [72, 73]. However, the economic development model that prioritizes efficiency has led to widening regional economic disparities and inequality in the supply of public goods, threatening the sustainable socioeconomic development in China [74]. Entering the 21st century, the orientation of economic policy has shifted from efficiency priority to a balanced concern for efficiency and fairness. Therefore, the Chinese government proposed a strategy of coordinating regional development in 2003, aiming to combine the advantages of capital, technology, talents and information in the eastern region with the advantages of resources, markets and labour in the central and western regions to form a regional economic pattern characterized by complementary advantages, distinctive features and coordinated development. Subsequently, a series of biased policies, such as the revitalization of the Northeast, the rise of central China, Chengdu-Chongqing economic circle, and the high-quality development of the Yellow River Basin, were introduced. As a result, the regional division of labour and cooperation system built on comparative advantages has been continuously improved, and the regional gap has been continuously reduced [75].

Although regional economic disparities in China have narrowed over the past few decades, economic inequality has not been fundamentally reversed, and new problems such as the solidification of regional disparities have even emerged [76, 77]. In line with the goals and requirements of the national RCD strategy, the regional location should be fully considered, and the comparative advantages of the region should be brought into play to develop targeted and differentiated macro-regional policies, improving the accuracy and effectiveness of financial, industrial, land, talent and other policies. Specifically, the eastern China should actively promote the transfer of industries to the central and western regions to drive the development of the central and western China, and take the lead in achieving structural balance among the three industries and be cautious of excessive de-industrialization. The central China should give full play to its advantages in transportation and grain production, further construct a new modern infrastructure network, and achieve all-round opening by amplifying its advantages of vertical and horizontal connectivity, while focusing on the development of advanced manufacturing and modern agriculture. The western China should enhance its capacity for sustainable development, focus on solving the two key issues of infrastructure support and ecological protection, and fully leverage the resource advantages of the northwest region and the industrial advantages of the southwest region to shape its core competitiveness. The northeastern China should aim to effectively enhance its development vitality, endogenous power and comprehensive competitiveness, deepen institutional reform, promote industrial structure optimization and adjustment, and facilitate high-quality development with innovation and entrepreneurship. In addition, it is necessary to develop macro-regional policies for the development of specific regions. For example, the government should actively promote the development of the Yangtze River Economic Belt by relying on the Yangtze River waterway and help the high-quality development of advantageous industries along the river; the construction of the "Belt and Road" should be accelerated to strengthen the infrastructure support in western China and promote the development of the border areas.

## 5 Conclusion

Since the founding of the People’s Republic of China in 1949, the country has undergone a major economic transformation, especially since the introduction of the reform and opening-up policy in 1978 [77]. Despite this progress, China still faces the challenge of economic inequality arising from spatial heterogeneity and unbalanced development strategies. This study aims to reveal the regional inequality of China’s economy in 1978–2018 and explores its stages and characteristics to guide the implementation of RCD strategy in the new era. The study found that China’s economy grew rapidly during this period, with an average annual growth rate of 9.4%. The secondary and tertiary sectors grew significantly faster than the primary sector, and the tertiary industry gradually took a dominant position in China’s industrial structure. Spatially, the share of GDP in the central and western China remained relatively stable, while that in the eastern and north-eastern China showed an increasing and decreasing trend, respectively. The CG analysis of GDP and the added-value of the three industries showed that most of the CGs during the period were distributed in the eastern and southern part of Henan Province, and moved southwest as a whole. According to the spatial heterogeneity analysis of economic inequality, China’s economic growth from 1978 to 2018 could be divided into four stages, namely, economic inequality led by institutional reform (1978–1991), market mechanisms (1992–2003), regional coordination (2004–2012), and socioeconomic transformation (2013–2018). This process reflects the implementation of Deng Xiaoping’s idea of “two big pictures” and highlights how to balance efficiency and equity in regional economic development.

Although the analysis results with GDP as the only criterion are somewhat different from the actual situation, GDP, as the internationally recognized and most widely used indicator, reflects the level of regional economic development to a considerable extent and is the most effective tool and important indicator for understanding the macroeconomic operation of a country or region. Of course, an in-depth analysis with the help of interregional economic linkages can reveal more comprehensively the characteristics of China’s economic inequality at different stages. This deserves further research. Ultimately, regional economic policy is about managing and coordinating the relationships between different actors [78]. Future research on deepening RCD needs to strengthen the study of government-market-individual interaction mechanisms, so as to improve the relevance and effectiveness of regional policies, reduce regional economic disparities, and help achieve the 2030 sustainable development goals.
